# The relationship between pitching parameters and release points of different pitch types in major league baseball players

**DOI:** 10.3389/fspor.2023.1113069

**Published:** 2023-04-24

**Authors:** Yasuhiro Hashimoto, Tomoyuki Nagami, Shinji Yoshitake, Hiroki Nakata

**Affiliations:** ^1^ Faculty of Sustainable System Sciences, Osaka Metropolitan University, Osaka, Japan; ^2^ College of Liberal Arts and Sciences, Kitasato University, Sagamihara, Japan; ^3^ Faculty of Engineering, Nara Women's University, Nara, Japan

**Keywords:** baseball, big data, release speed, spin rate, pitcher

## Abstract

**Objectives:**

The purpose of this study was to deepen our understanding of pitches and to obtain basic knowledge about pitches by comparing 4-seam and other pitches in Major League Baseball (MLB).

**Methods:**

We analyzed big data for 1,820 professional baseball pitchers of MLB on release speed, spin rate, release point 3D coordinates (X, Y, and Z axes), amount of change for 4-seam, and seven changing ball types (sinker, slider, changeup, cutter, curve, split finger, and knuckle curve), using PITCHf/x and TrackMan. We also evaluated three relationships: (1) between the release points and the ball types of pitch; (2) between the amount of change in the ball and the release speed; and (3) between the release speed and the spin rate.

**Results:**

The release speed was significantly slower in seven changing ball types than in the 4-seam (*p* < 0.01, respectively). The spin rate and the amount of change (ΔX and ΔZ) were significantly different between 4-seam and seven changing ball types (*p* < 0.01, respectively). Release point 3D coordinates (X, Y, and Z axes) were significantly different between 4-seam and slider, cutter, and curve (*p* < 0.01, respectively). Based on these findings, the eight pitch types were mainly divided into three groups: 4-seam, curve, and off-speed pitch types.

**Conclusion:**

Seven changing ball types included specific characteristics for each parameter. The correspondence among the release speed, ΔX, and ΔZ at the 3D coordinates is an arch with 4-seam as the apex. Our results suggest an effective strategy for changing the release point and displacement of a ball's trajectory to improve the performance of baseball pitchers.

## Introduction

Baseball pitchers often increase ball speed and use a variety of changing balls to prevent hard hits by batters. Pitchers have control over the speed, ball spin rates, and direction of the spin axis. These factors are combined to modulate the direction and magnitude of ball displacement (1). Previous studies on pitching by baseball pitchers included examination of the relationship between the pitches and different parameters. For example, Jinji and Sakurai (2) reported that the angular speed (rotational speed) of the ball was proportional to the moving speed. We showed that the amount of lift force acting on the ball could be explained by the effective spin parameter (1). In addition, pitching kinematics have been extensively examined (3). For example, it has been reported that 4-seam (although previous studies described the corresponding ball type as 4-seam, fastball, or 4-seam fastball, among others, in this study, we will use the unified form “4-seam”) had a higher medial elbow torque than curve and changeup (4). Compared with those in changeups, fastballs, sliders, and curves, 4-seam require high shoulder and elbow strength and torque, increasing the injury risk (5). Regarding elbow varus torque, shoulder internal rotation torque, elbow proximal force, and shoulder proximal force, the fastball produced the greatest values, followed by the curveball and then the changeup (6). In addition, we (1) discovered that the effective spin parameter and the directions of the ball's linear and angular velocities could explain more than 90% of the variance in the relationship between the components acting in the left-right and up-down directions.

On the other hand, many studies have focused on the batter's perspective. In terms of ball speed and spin rate, Higuchi et al. (7) reported a decrease in the accuracy of the batter's swing, when the fastball's backspin deviated from the usual rate. As for the pitching motion and ball trajectory, some studies showed the batter's specific visual search strategy to evaluate the pitcher's motion, including viewing duration for the pitcher's arm (8) and body parts (9), and to examine the ball trajectory (10, 11). In general, it is said that a good batter can detect the habits of a pitcher. For example, this includes information that can be obtained before pitching, including the grab height when setting the ball and how the grab swells. If the release points differed greatly among ball types, the batter would be able to detect the pitcher's ball type from the release point information. Therefore, pitchers need to have a constant pitching form among the ball pitch types. In other words, since high-level pitchers have a constant pitching form, it is difficult for batters to predict which type of pitch will be thrown.

However, after a thorough literature search, few studies were found that included examining the statistical difference in release points among pitch types, even though many studies on baseball pitching have been published. Recently, Kusafuka et al. (12) showed that the 4-seam was pitched from different release points as well as speed and ball angle among seven pitchers. In their research, since even skilled professional pitchers have errors in each movement, they mainly used 4-seam to estimate the factors affecting throwing parameters. We also focused on the 4-seam in 11 collegiate and 11 professional pitchers, reporting a correlation (*r* = 0.477, *p* < 0.05) between ball speed and spin rate (13). However, our previous study did not analyze the data on the release points. As per the limitations of these studies, other ball types such as sliders, changeups, and curves were not examined. If their methods had been applied to changing-balls for changing the ball's arrival position compared with that of 4-seam, the throwing strategy, including the release point and the spin rate, may be significantly different. Moreover, the number of pitchers was small. As for the reason, we assumed that the pitch types markedly differed among all pitchers. That is, “pitcher A” can throw the 4-seam, sinker, slider, and changeup, while “pitcher B” can pitch the 4-seam, cut, split finger, and curve. Therefore, it would be difficult to clarify the difference in the pitching data associated with the pitch types of many pitchers. Indeed, our previous study showed data on ball characteristics of 4-seam from seven pitchers: 2-seam from five pitchers, slider from five pitchers, forkball from three pitchers, curveball from one pitcher, cut from one pitcher, and changeup from one pitcher (1). In this research, large letters (E, M, and I) were inscribed on the surfaces of the balls to analyze ball spin, and then 3D motion analysis was performed to accurately measure the speed and spin rate of each ball. However, it is not realistic to obtain data on many top-level pitchers under these conditions. Therefore, the generalization of these results presents certain limitations. In addition, some studies showed the differences in pitching form, including release speed and release point 3D coordinates, among ball pitch types, using 3D motion analysis and wearable devices. However, the number of pitchers was limited (29 pitchers: Dun et al. (5), 33 pitchers: Nissen et al. (14), 18 pitchers: Escamilla et al. (4), 37 pitchers: Makhni et al. (15)).

Based on these research backgrounds, the present study used big data from PITCHf/x (Sportvision, Chicago, IL) and TrackMan (TrackMan, Inc., Stamford, CT) in Major League Baseball (MLB) games. MLB used PITCHf/x until 2015 and TrackMan after 2016 to measure data. The Baseball Savant containing these data provided open data on MLB, containing over 400,000 balls. In this sense, some previous studies have already used this system (16–19). For example, Glanzer et al. (19) investigated the relationship between variability in pitching kinematics and consistency in pitch location using PITCHf/x. They analyzed the data on 47 baseball pitchers throwing 10 full-effort fastballs with 20 kinematic parameters. Whiteside et al. (16) focused on 7,600 pitches from 199 starting MLB pitchers and analyzed the performance variables that influenced the pitching results. They reported some variables, including the maximum speed of the ball, consistent spatial release location, and various ball speeds.

Furthermore, PITCHf/x is widely used in research on big data in baseball (18, 20), suggesting the usefulness of big data from the Baseball Savant in clarifying the pitching performance in baseball games. Especially, the immediate feedback is available to the pitcher (21). Feedback effectiveness has been demonstrated to improve pitching performance in some studies (22–24). PITCHf/x and TrackMan provide data regarding release speed, spin rate, release point 3D coordinates (X, Y, and Z axes), and amount of change. For example, if “pitcher A” wants to change the slider in the horizontal direction by 20 cm, he can easily check his performance after finishing the throw.

By applying big data from PITCHf/x and TrackMan in MLB, the present study focused on three relationships: (1) between the release points and the ball types of pitch; (2) between the amount of change in the ball and the release speed; and (3) between the release speed and the spin rate. The main reasons for clarifying these associations are: (1) to provide basic data for the question of whether the batter can distinguish the type of pitch by the position of the release point or not; (2) to discern the characteristics of the average ball type; and (3) to reveal how much a baseball player can vary the amount of change and the release speed of the ball in pitching. In terms of pitching ball comparisons, the data on 4-seam was primarily used because it is thrown by most of MLB pitchers. Changing-balls involved sinker, slider, changeup, cutter, curve, split finger, and knuckle curve. We hypothesized that the release points differed depending on the ball type, because each ball type includes specifical characteristics, such as different release speed, spin rate, and spin axes. The significance of the present study is that we provide basic big data on top-level baseball players that has not been previously investigated. Evaluating how MLB players throw the ball can help us to determine what kind of ball a general baseball pitcher should throw and what kind of ball a general baseball batter should hit. Our data should help many baseball players, coaches, parents, and strength and conditioning professionals to achieve their desired goals.

The purpose of this study was to obtain basic knowledge about pitches by comparing 4-seam and other pitches in MLB. We hypothesized the following: 1) release speed, spin rate, and X, Y, Z, ΔX and ΔZ values for 4-seam and changing-balls are different, and 2) eight pitch types (4-seam, sinker, slider, changeup, cutter, curve, split finger, and knuckle curve) can be broadly divided into several types based on each parameter.

## Materials and methods

### Sample

The pitching data on MLB players were obtained from MLB.com *via* (25) “Baseball Savant” (26). We analyzed the data of 1,820 pitchers in the MLB official games from 2015 to 2021, excluding 2020 when the full season was not played. MLB used PITCHf/x until 2015 and TrackMan after 2016 to measure data. The data included the pitcher's name, team, pitch type, dominant hand, release point 3D coordinates, release speed, spin rate, and amount of change (ΔX and ΔZ). The data that lacked any of these factors as well as submarine pitchers (players with an average release point of 140 cm or less) were excluded from the overall averaging and analysis. If the same pitcher pitched for multiple years, the data from the most recent year were adopted.

In the 3D coordinate data, the X-axis was directed from the pitcher's plate to the third base in the right-handed pitcher. The Y-axis was directed from the pitcher plate to the home base. The Z-axis was directed from the pitcher's plate vertically upward. In order to unify the data for the right-handed pitcher, in the case of the left-handed pitcher, the data of X-axis were inversely calculated. For the amount of change in each ball type, the values of pfx_x and pfx_z were analyzed. This data was set as horizontal and vertical movement of the pitch compared to a theoretical pitch of the same speed with no spin-induced movement and expressed by ΔX and ΔZ, following previous studies (27, 28). The X-axis of ΔX is in the right direction as seen from the pitcher, and the Z-axis of ΔZ is in the vertical upward direction. Finally, the present study focused on the data on release speed, spin rate, release point 3D coordinates (X, Y, and Z axes), and amount of change (ΔX and ΔZ) for 4-seam, and seven changing ball, including sinker, slider, changeup, cutter, curve, split finger, and knuckle curve. These parameters were registered in the Baseball Savant database, and widely used to show the pitching motion and physics of a pitched ball. We checked all data before analyzing, and excluded the data that threw 50 or fewer balls per year (ex., knuckles).

Table 1 shows the number of samples and the mean number of pitches per pitcher for each type of pitch. In the present study, the number of samples differed for each ball type, and pitchers who threw 50 or more balls per year with the relevant ball type were analyzed.

**Table 1:** Number of data for each type of pitch.

Pitch. type		Number of pitches per pitcher
	*n*	Mean (SD)
4-seam	1363	324 (300)
Sinker	705	275 (263)
Slider	927	224 (176)
Changeup	734	183 (149)
Cut	300	252 (209)
Curve	604	183 (154)
Split finger	91	240 (193)
Knuckle curve	91	257 (200)

The left line indicates the number of samples for each type of pitch to be analyzed. The right line shows the numbers of pitches per pitcher with standard deviation (SD).

**Figure 1:**
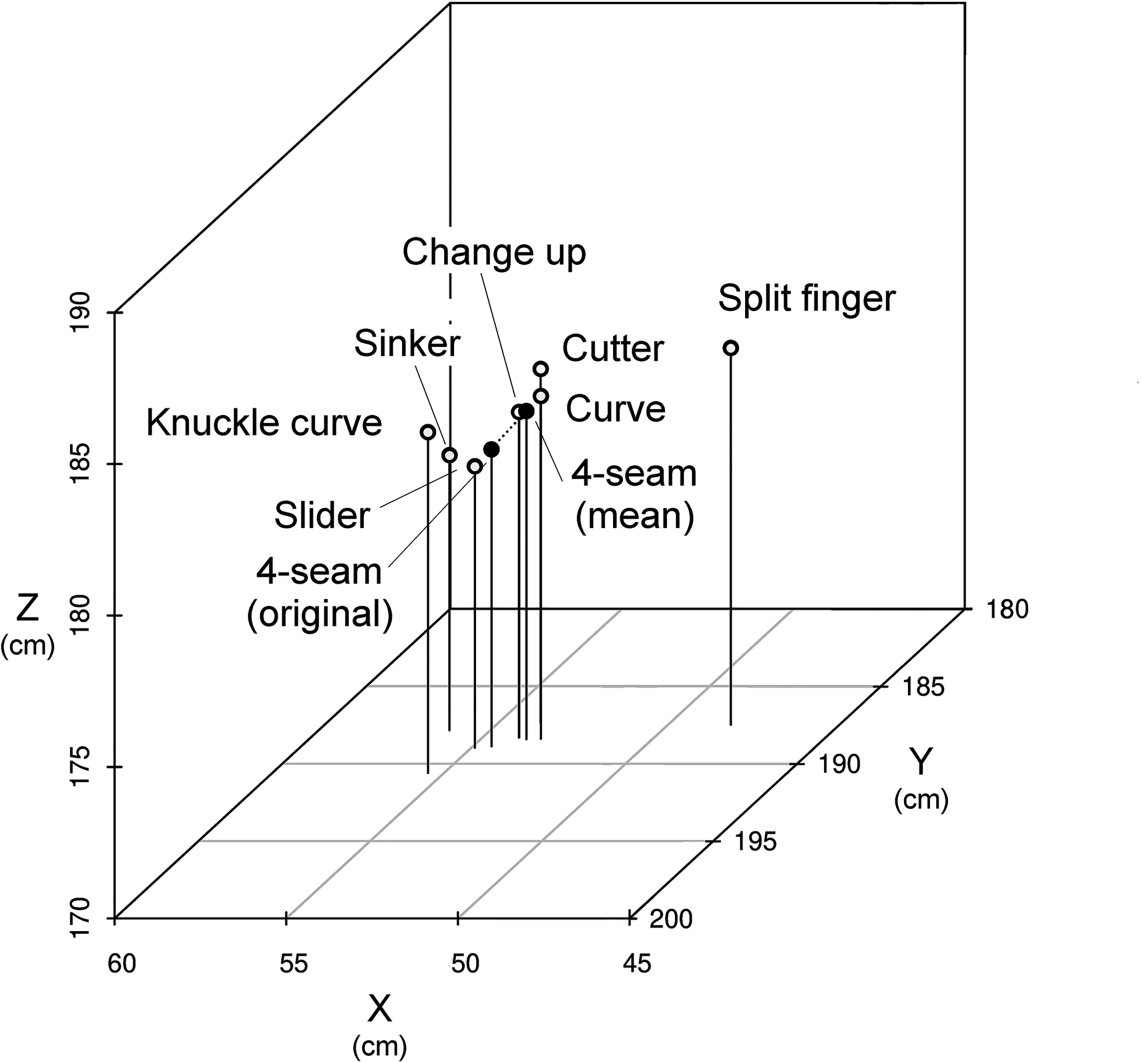
Difference in release point of 4-seam according to ball type. Data in the white circle with the name of each ball type indicate the 4-seam data of the pitcher who threw each ball type. 4-seam (original) shows the average data of pitchers (*n* = 1363) who threw more than 50 4-seam balls a year between 2015 and 2021, excluding 2020. 4-seam mean demonstrates the average value of the 3D coordinates (X, Y, and Z axes) for each ball type (data in seven white circles).

To clarify the difference in each variable data among pitch types, we set the average data of 4-seam as the origin, because the number of samples for 4-seam was markedly large (i.e., most pitchers threw 4-seam). Then, we corrected the data on seven changing ball types, based on the average data of 4-seam. For example, the release points of the 4-seam differ between pitchers who can throw a sinker or a slider. The release point of the 4-seam for pitchers who can throw a slider is more of a side throw. In other words, the data on 4-seam are affected by the changing ball types that pitchers can throw. Therefore, in order to clarify the difference in the release point among ball types, it was necessary to correct the data on 4-seam corresponding to each changing ball type. Figure 1 shows the 4-seam release points for each ball type. The 4-seam (original) indicates the average data of pitchers (*n* = 1363) who threw more than 50 4-seam balls per year between 2015 and 2021. 4-seam mean shows the average value of 4-seam for each ball type (sinker, slider, changeup, cutter, curve, split finger, and knuckle curve). The 4-seam data regarding release speed, spin rate, and release point 3D coordinates of pitchers who threw more than 50 pitches of each ball (sinker, slider, changeup, cutter, curve, split finger, and knuckle curve) in the past year were averaged. That is, data from more than 50 pitches in two pitch types (each ball type and 4-seam) were included in the analysis. At the time, 4-seam was used as the 4-seam mean for each pitch type. The 4-seam mean was used as the reference point for the corrected value. The collected average value of 4-seam was as follows: speed = 149.30 km/h, spin = 2241.15 r/min, X = 53.67 cm, Y = 188.42 cm, Z = 180.83 cm, ΔX = 19.54 cm, and ΔZ = 40.02 cm.

### Data analysis

To compare release points and amount of change among ball types, we subtracted the average value of 4-seam depending on seven changing ball types (sinker, slider, changeup, cutter, curve, split finger, and knuckle curve) from each basic 4-seam value in each changing ball type, and added the value of the corresponding ball type (i.e., we referred to this as “corrected value”). This process was performed on the X, Y, and Z axes for release point, and ΔX and ΔZ for amount of change. By making this correction, it is possible to make a relative comparison in release points and amount of change between 4-seam and seven changing ball types. Then, the differences in each variable data between 4-seam and seven changing ball types were statistically examined by the paired *t*-test. Furthermore, we analyzed the bivariate correlative relationship between each parameter using the Pearson product-moment correlation coefficient. In order to calculate the correlation between ball types, the average value of each ball type was used. Direct distance is calculated by Formula 1 for 2D and Formula 2 for 3D. SPSS Ver. 26 for Windows (IBM) was used for statistics. The values were expressed as mean ± standard deviation (SD), and the significance level was set at *p* < 0.05.

## Results

Table 2 presents the mean values of release speed, spin rate, release point 3D coordinates (X, Y, and Z axes), and amount of change (ΔX and ΔZ) for pitch types and each 4-seam corresponding to each ball type. Compared to 4-seam, seven changing-ball types differed in spin rate (*p* < 0.01, respectively). The fastest release speed was 149.54 ± 1.86 km/h for 4-seam, and the slowest was 126.18 ± 2.39 km/h for curve. The 4-seam mean corresponding to each ball type was significantly different in all ball types (*p* < 0.01, respectively), indicating that the release speed was higher in the 4-seam mean than in each ball type. Curve had the highest spin rate at 2449.57 ± 156.61 r/min, while split finger had the lowest at 1406.25 ± 101.16 r/min. The spin rate was significantly higher in the 4-seam mean than in sinker, changeup, and split finger (*p* < 0.01, respectively), while it was lower in the 4-seam mean than in slider, cutter, curve, and knuckle curve (*p* < 0.01, respectively).

**Table 2:** Mean values of release speed, spin rate, release point 3D coordinates (X, Y, and Z axes), and amount of change (ΔX and ΔZ) for pitch types and 4-seam mean corresponding to each ball type.

Release speed (km/h)	Mean	4-seam mean	Corrected value
4-seam	149.54 (1.86)		149.30
Sinker	148.55 (1.92)	149.16 (1.89)**	148.69
Slider	135.81 (2.36)	149.97 (1.87)**	135.38
Changeup	136.64 (2.15)	149.05 (1.92)**	137.13
Cutter	141.94 (2.14)	148.54 (1.88)**	142.94
Curve	126.18 (2.39)	149.01 (1.90)**	126.71
Split finger	137.09 (2.21)	150.06 (1.87)**	136.57
Knuckle curve	128.40 (2.37)	149.31 (1.96)**	128.63
Spin rate (r/min)	Mean	4-seam mean	Corrected value
4-seam	2242.35 (103.77)		2241.15
Sinker	2156.08 (107.99)	2233.48 (103.36)**	2163.75
Slider	2344.54 (179.73)	2252.72 (104.51)**	2332.97
Changeup	1730.77 (159.19)	2226.25 (103.47)**	1745.67
Cutter	2312.39 (132.77)	2251.84 (106.64)**	2301.70
Curve	2449.57 (156.61)	2245.66 (103.74)**	2445.07
Split finger	1406.25 (240.53)	2219.85 (101.16)**	1427.55
Knuckle curve	2411.00 (138.02)	2258.27 (104.66)**	2393.89
X (cm)	Mean	4-seam mean	Corrected value
4-seam	54.46 (6.35)		53.67
Sinker	58.89 (6.57)	56.19 (6.67)**	56.37
Slider	58.74 (6.30)	54.90 (6.52)**	57.50
Changeup	57.28 (6.21)	53.93 (6.77)**	57.01
Cutter	56.53 (6.31)	53.77 (6.56)**	56.43
Curve	54.69 (6.21)	53.26 (6.47)**	55.10
Split finger	49.64 (6.16)	48.17 (6.47)**	55.15
Knuckle curve	56.31 (6.63)	55.47 (6.96)	54.51
Y (cm)	Mean	4-seam mean	Corrected value
4-seam	188.88 (5.96)		188.42
Sinker	187.93 (6.36)	187.84 (6.33)	188.51
Slider	182.85 (5.85)	188.97 (5.98)**	182.31
Changeup	188.50 (6.55)	188.32 (6.12)	188.61
Cutter	184.39 (5.75)	187.36 (6.03)**	185.45
Curve	177.93 (7.21)	188.39 (6.12)**	177.96
Split finger	184.00 (6.27)	187.49 (5.98)**	184.93
Knuckle curve	181.40 (6.32)	190.60 (5.96)**	179.23
Z (cm)	Mean	4-seam mean	Corrected value
4-seam	179.80 (3.58)		180.83
Sinker	176.65 (3.94)	179.08 (3.83)**	178.39
Slider	177.84 (3.70)	179.29 (3.67)**	179.38
Changeup	178.61 (3.72)	180.75 (3.74)**	178.68
Cutter	180.52 (3.67)	181.68 (3.68)**	179.67
Curve	183.24 (3.78)	181.31 (3.68)**	182.77
Split finger	181.97 (3.55)	182.44 (3.54)	180.36
Knuckle curve	182.84 (3.58)	181.25 (3.81)**	182.41
ΔX (cm)	Mean	4-seam mean	Corrected value
4-seam	19.56 (5.90)		19.54
Sinker	36.59 (5.64)	19.58 (5.98)**	36.55
Slider	−12.98 (6.51)	19.53 (5.92)**	−12.97
Changeup	33.46 (6.57)	20.09 (5.94)**	32.91
Cutter	−4.36 (6.02)	17.78 (6.01)**	−2.60
Curve	−21.15 (6.82)	18.71 (5.99)**	−20.32
Split finger	26.55 (7.80)	21.20 (5.63)**	24.90
Knuckle curve	−19.08 (6.84)	19.90 (5.83)**	−19.44

ΔZ (cm)	Mean	4-seam mean	Corrected value
4-seam	40.52 (5.32)		41.02
Sinker	27.20 (5.99)	39.57 (5.58)**	28.66
Slider	6.23 (7.27)	40.47 (5.29)**	6.79
Changeup	21.27 (7.36)	40.98 (5.44)**	21.32
Cutter	22.79 (7.20)	41.48 (5.70)**	22.34
Curve	−20.86 (6.98)	41.27 (5.46)**	−21.10
Split finger	13.71(8.76)	42.13(5.12)**	12.60
Knuckle curve	−23.73(6.86)	41.29(5.38)**	−23.99

“4-seam mean” indicates the data on 4-seam corresponding to each changing ball type. “Corrected value” means the value calculated using Formula 3. The difference was determined using the paired *t*-test between the 4-seam mean corresponding to each changing ball type and corrected values. Data are expressed as mean with standard deviation (SD).

** *p* < 0.01.

**Figure 2:**
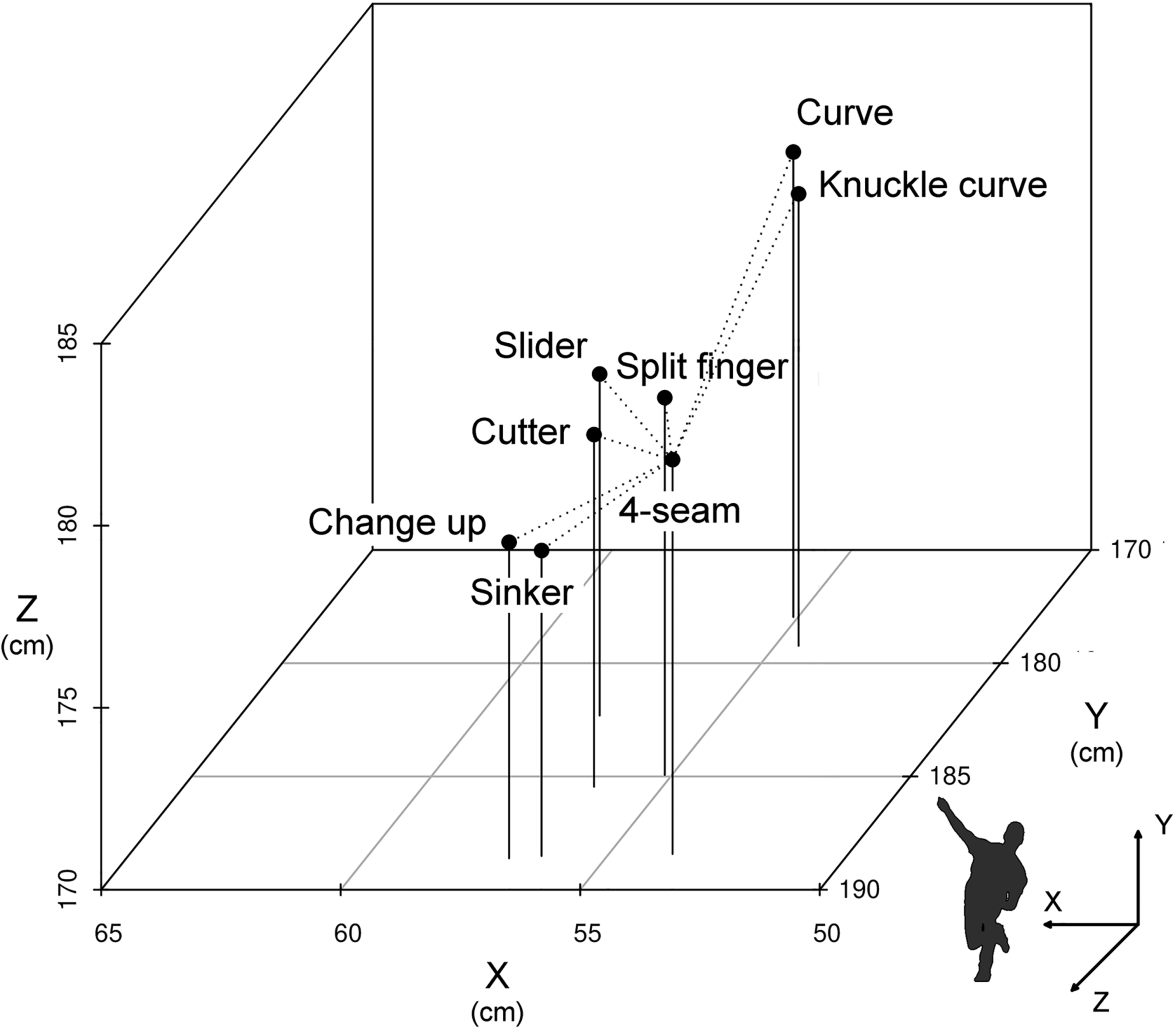
3D coordinates of the release point for each ball type. 4-seam mean was used as the reference point for corrected values.

Figure 2 shows the 3D coordinates of the release point for each ball type. The 3D coordinates of curve were the farthest from those of 4-seam (direct distance = 10.73 cm; X = 1.43 cm, Y = −10.46 cm, Z = 1.94 cm), while the 3D coordinates of sinker were the closest to those of 4-seam (direct distance = 3.64 cm; X = 2.70 cm, Y = 0.08 cm, Z = −2.44 cm). Based on Figure 2, the release points were summarized as follows: all ball types were right lateral to 4-seam, and curve, knuckle curve, slider, split finger, and cutter were lower than 4-seam. Curve and knuckle curve were higher than 4-seam. Changeup and sinker were posterior than 4-seam.

**Figure 3:**
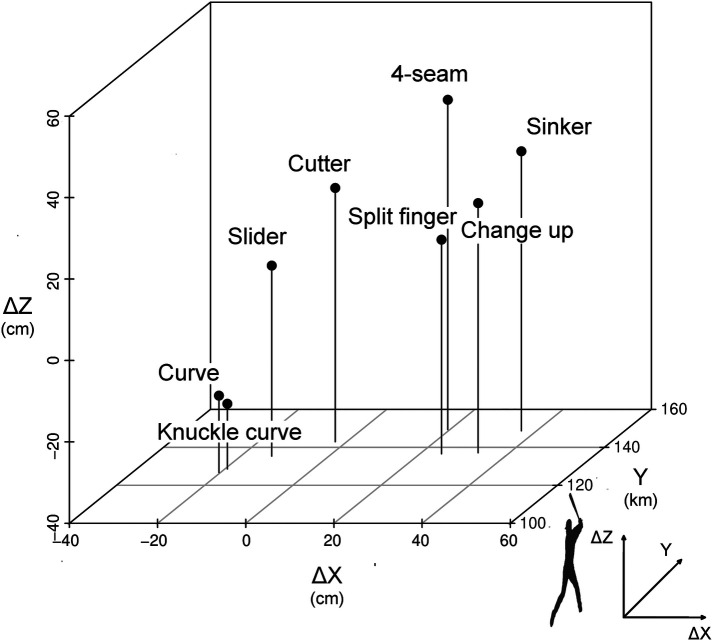
3D coordinates of ΔX and ΔZ and release speed. ΔX, release speed, and ΔZ are shown in X, Y, and Z axes, respectively. The distance between 4-seam and each type of pitch is as follows: sinker, M = 21.33 cm; slider, M = 47.57 cm; changeup, M = 24.23 cm; cutter, M = 29.29 cm; curve, M = 74.23 cm; split finger, M = 29.41 cm; and knuckle curve, M = 76.23 cm.

Figure 3 shows the 3D coordinates of ΔX, ΔZ, and release speed. The amount of change of knuckle curve was the farthest from that of 4-seam (direct distance = 75.80 cm; ΔX = −38.98 cm, ΔZ = −65.01 cm), and the amount of change of sinker was the closest to that of 4-seam (direct distance = 21.02 cm; ΔX = 17.00 cm, ΔZ = −12.37 cm). The value of ΔZ in slider was the closest to the ΔZ origin among all changing balls (i.e., ΔZ = 6.79 cm, Table 2). This value was physically the closest to free fall.

Table 3 presents the correlation coefficient between release speed and ΔX and ΔZ from all pitch types. With regard to the correlation between each parameter, significant relationships were observed between *Δ*X and ΔZ (*r* = 0.77, *n* = 8, *p* < 0.05) and between ΔZ and release speed (*r* = 0.94, *n* = 8, *p* < 0.01). No significant relationship was found between ΔX and release speed (*r* = 0.70, *n* = 8, *p* = 0.06). Based on Figure 3, ΔX and ΔZ values are summarized as follows: for ΔX, sinker, changeup, and split finger by pitchers were right lateral to 4-seam, and curve, knuckle curve, slider, and cutter were left lateral to 4-seam. The ΔZ was lower for all changing-ball types than for 4-seam.

**Table 3:** Correlation coefficient between release speed and ΔX and ΔZ from all pitch types.

	Release speed	ΔX
ΔX	0.70	–
ΔZ	0.94**	0.77*

* *p* < 0.05.

** *p* < 0.01.

Table 4 shows the correlation coefficient between release speed, release point 3D coordinates (X, Y, and Z axes), spin rate, and amount of change (ΔX and ΔZ) for pitch type. The 4-seam correlation coefficient was *r* = −0.39–0.48. The sinker correlation coefficient was *r* = −0.40–0.57. The slider correlation coefficient was *r* = −0.49–0.50. The changeup correlation coefficient was *r* = −0.30–0.45. The cutter correlation coefficient was *r* = −0.44–0.51. The curve correlation coefficient was *r* = −0.44–0.29. The split finger correlation coefficient was *r* = −0.33–0.49. The knuckle curve correlation coefficient was *r* = −0.50–0.30.

**Table 4:** The r value of correlation coefficient among release speed, release point 3D coordinates (X, Y, and Z axes), spin rate, and amount of change (ΔX and ΔZ) for pitch type.

4-seam	Spin	X	Y	Z	ΔX	ΔZ
Speed	0.31**	−0.06**	0.13**	0.12**	0.08**	0.05
Spin		0.02	−0.02	0.00	−0.12**	0.19**
X			0.08**	−0.36**	0.26**	−0.39**
Y				−0.11**	−0.12**	0.00
Z					−0.29**	0.48**
ΔX						−0.15**
Sinker	Spin	X	Y	Z	ΔX	ΔZ
Speed	0.35**	−0.14**	0.12**	0.24**	0.06	0.18**
Spin		−0.03	−0.07	0.05	−0.03	0.20**
X			0.10**	−0.37**	0.23**	−0.40**
Y				−0.13**	−0.09**	−0.01
Z					−0.26**	0.57**
ΔX						−0.37**
Slider	Spin	X	Y	Z	ΔX	ΔZ
Speed	0.00	−0.18**	0.07**	0.29**	0.50**	0.32**
Spin		0.05	−0.06	−0.06	−0.49**	−0.35**
X			0.05	−0.37**	−0.12**	0.00
Y				−0.10**	−0.10**	0.00
Z					0.24**	−0.10**
ΔX						0.37**
Changeup	Spin	X	Y	Z	ΔX	ΔZ
Speed	0.14**	0.03	0.09**	−0.03	0.21**	−0.17**
Spin		0.05	−0.04	−0.07	0.42**	0.14**
X			0.05	−0.30**	0.25**	−0.29**
Y				−0.13**	0.00	0.03
Z					−0.27**	0.45**
ΔX						−0.27**
Cutter	Spin	X	Y	Z	ΔX	ΔZ
Speed	0.14**	−0.06	0.12**	0.07	0.17**	0.36**
Spin		0.00	−0.04	−0.10	−0.46**	−0.27**
X			0.01	−0.27**	0.10	−0.11
Y				−0.11**	−0.15**	−0.03
Z					−0.05	0.12**
ΔX						0.51**
Curve	Spin	X	Y	Z	ΔX	ΔZ
Speed	0.14**	−0.04	0.05	−0.09**	0.29**	0.29**
Spin		0.02	−0.12**	−0.09**	−0.44**	−0.28**
X			−0.02	−0.34**	−0.15**	0.20**
Y				−0.11**	−0.02	−0.15**
Z					0.26**	−0.23**
ΔX						0.15**
Split finger	Spin	X	Y	Z	ΔX	ΔZ
Speed	0.30**	0.06	0.28**	−0.10	0.04	−0.03
Spin		0.24**	−0.21	−0.20	0.49**	0.45**
X			0.05	−0.26**	0.32**	0.06
Y				−0.23**	−0.25**	−0.15
Z					−0.33**	0.10
ΔX						0.27**
Knuckle curve	Spin	X	Y	Z	ΔX	ΔZ
Speed	0.19	−0.06	0.19	−0.21**	0.28**	0.12
Spin		−0.14	0.10	−0.15	−0.39**	−0.50**
X			0.07	−0.37**	−0.01	0.29**
Y				−0.17	0.09	−0.18
Z					0.30**	−0.25**
ΔX						0.23**

** *p* < 0.01.

**Figure 4:**
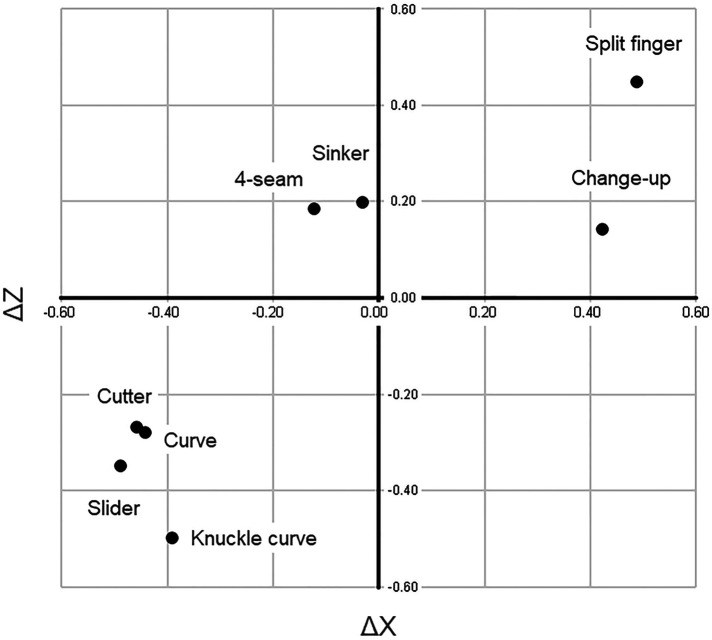
Relationship between spin rate and ΔX and between spin rate and ΔZ, as shown by correlation coefficient.

Figure 4 shows the relationship in correlation coefficient between spin rate and ΔX, and spin rate and ΔZ.

Compared with those of 4-seam, the values in ΔX were negative for curve, knuckle curve, slider, and cutter and positive for sinker, changeup, and split finger (Table 2). In addition, the correlation analysis showed a positive relationship in cutter and slider between ΔX and ΔZ (*r* = 0.51, *p* < 0.01; *r* = 0.37, *p* < 0.01, respectively) and a negative relationship in sinker between ΔX and ΔZ (*r* = −0.37, *p* < 0.01) (Table 4). Although the names of cutter and slider are different as pitch types, the correlation coefficient values were similar. Therefore, we considered that it was difficult for cutter, slider, and sinker pitchers to show their individuality compared with other types of pitchers. Slider, cutter, curve, knuckle curve, and 4-seam showed a negative correlation between spin rate and ΔX (*r* = −0.49, *p* < 0.01; *r* = −0.46, *p* < 0.01; *r* = −0.44, *p* < 0.01; *r* = −0.39, *p* < 0.01; *r* = −0.12, *p* < 0.01, respectively). Contrastingly, split finger and changeup showed a positive correlation between spin rate and ΔX (*r* = 0.49, *p* < 0.01; *r* = 0.42, *p* < 0.01, respectively). The knuckle curve, slider, curve, and cutter showed a negative correlation between spin rate and ΔZ (*r* = −0.50, *p* < 0.01; *r* = −0.35, *p* < 0.01; *r* = −0.28, *p* < 0.01; *r* = −0.27, *p* < 0.01, respectively).

## Discussion

The purpose of the present study was to obtain basic knowledge about pitches by comparing 4-seam and other pitches in MLB. The present study included more than 10 million data on MLB pitchers that have been published in the Baseball Savant database. We analyzed release speed, spin rate, release point 3D coordinates, and amount of change for 4-seam and seven changing ball types. We mainly set the data for 4-seam and focused on the differences in characteristics between 4-seam and seven changing ball types. We demonstrated that these pitching parameters significantly differed among the changing ball types. Our results suggested an effective strategy for changing the release point and displacement of a ball's trajectory to improve the performance of baseball pitchers. Recently, Rapsodo (29) has widely been used as a training tool that provides immediate feedback on ball parameters to pitchers and coaches. Our data propose a reference value for baseball pitchers to train with.

The mean value of release speed for 4-seam in the present study was 149.54 ± 1.86 km/h (Table 2). Our result was clearly higher than previous studies: 106.20 ± 7.56 km/h (14), 117.36 ± 7.92 km/h (12), 121.68 ± 6.12 km/h (2), and 135.72 ± 4.32 km/h (13). Similarly, the mean value of spin rate for 4-seam in the present study was 2244.95 ± 99.90 r/min (Table 2). This result was also higher than previous studies: 1740 ± 168 r/min (12), 1884 ± 162 r/min (13) and 2058 ± 210 r/min (2). We assumed the differences were in the participants. We showed data on MLB players using big data from PITCHf/x and TrackMan, while these previous studies recruited mainly amateur players. Thus, our data would be useful as an index in the world's top-level pitchers.

On the other hand, positive correlations between 4-seam ball speed and spin rate were observed in previous studies [*r* = 0.905 (2); *r* = 0.477 (13)] as well as in the present study (*r* = .340, *p* < 0.01). Therefore, the relationship between 4-seam ball speed and spin rate might not be dependent on the player's level. Bahill and Baldwin (30) used computer simulations to estimate the spin rate of each pitch type for MLB pitchers and showed approximately 1200 r/min for 4-seam, 1400 r/min for slider, 2000 r/min for curve, and 400 r/min for changeup. In the present study, the spin rate was 2242.35 r/min for 4-seam, 2344.54 r/min for slider, 2449.57 r/min for curve, and 1738.67 r/min for changeup, respectively (Table 2). These values were also 1.87 times for 4-seam, 1.67 times for slider, 1.22 times for curve, and 4.33 times for changeup, higher than those from Bahill and Baldwin (30). This discrepancy might be related to a difference in estimated and measured values.

Data regarding the correlation between release speed and ΔX and ΔZ indicate that the release speed is more directly related to the fall of the ball. In addition, the values of release speed, spin rate, ΔX, and ΔZ were significantly different between the 4-seam mean and all changing balls (Table 2). This suggests that the characteristics of 4-seam are clearly different from those of other ball types. This notion is supported by data in Figure 3. The correspondence among the release speed, ΔX, and ΔZ at the 3D coordinates is an arch with 4-seam as the apex (Figure 3). The 4-seam and the apex of the coordinates had the highest release velocity and ΔZ values among all ball types. Then, we considered interpreting the data on ΔX, which indicates horizontal movement of pitch. The curve, knuckle curve, slider, and cutter values were significantly more negative than the 4-seam values (Table 2). Even if similar negative values in ΔX were shown, the pitching strategy might differ among pitch types. That is, the release point was clearly higher in curve than in 4-seam, but no significant difference was observed between split finger and 4-seam. Alternatively, the release point was lower in slider and cutter than in 4-seam (Figure 2). In straight-line distance, the release points of the 4-seam and changing-balls ranged from 3.64 cm (sinker) to 10.73 cm (curve). Fleisig et al. (31) investigated the kinetic parameters of 4-seam, curve, slider, and changeup, where they reported differences between 4-seam and curve. Furthermore, Smidebush et al. (32) showed that muscle activities of the lower and upper extremities differed between 4-seam and curve. The release points used in this study were the results induced by a combination of kinematic features, which supported the results in these previous studies. Future research must be performed to determine whether a batter understands the difference in the pitcher release point depending on the ball type.

Nissen et al. (14) conducted motion analyses of some pitch types, showing a significant difference in the elbow varus moment between 4-seam (59.6 ± 16.4°) and curve (54.1 ± 16.1°). Their data support the findings of the present study, indicating that the wrist may be more posterior in curve than in 4-seam, when the elbow is set at the same point. Bitzer (33) described the split finger as “more of a pronounced drop as it nears the plate, as if there was no lift acting on it.” In this study, the split finger was also the ball type that was nearly the closest to free fall.

Based on the spin rate and correlation analysis between ΔX and ΔZ, ball pitches were divided into three main groups (Figure 4). The first was the 4-seam group (4-seam and sinker). The correlation coefficients were negative between spin rate and ΔX and positive between spin rate and ΔZ. The second was the curve group (curve, knuckle curve, slider, and cutter). The correlation coefficients were negative between spin rate and ΔX, and between spin rate and ΔZ. The third was the off-speed pitch group (changeup and split finger). The correlation coefficients were positive between spin rate and ΔX and between spin rate and ΔZ. In addition, release speed and spin rate were significantly correlated in sinker, 4-seam, and changeup (*r* = 0.35, *p* < 0.01; *r* = 0.31, *p* < 0.01; *r* = 0.30, *p* < 0.01, respectively) (Table 4). In other words, significant relationships were observed in the 4-seam and off-speed pitch groups, but not in the curve group. Furthermore, as for the correlation with the release speed in each pitch type, slider showed a positive correlation between the release speed and ΔX (*r* = 0.50, *p* < 0.01). Cutter and slider showed a positive correlation between release speed and ΔZ (*r* = 0.36, *p* < 0.01, *r* = 0.32, *p* < 0.01, respectively) (Table 4). These data indicate that release speed and ΔX or ΔZ were significantly correlated in the curve group but not in the 4-seam and off-speed pitch groups.

As summarized in our data, ball types can be classified into three main groups. The first is the 4-seam group (4-seam and sinker). The characteristics that mainly define this group are the release speed, which is the fastest, the positive value of ΔX, and the highest value of ΔZ. The high correlation coefficient between release speed and spin rate is also a specific characteristic of this group. The second is the curve group (curve, knuckle curve, slider, and cutter). These ball types are characterized by falling while bending in the opposite direction to that of the dominant hand, with this trend being especially noticeable in sliders. In addition, ΔX and ΔZ show negative values, while ΔX and ΔZ are correlated. One of the main features is that the amount of change increases as the release speed decreases. The third is the off-speed pitch group (changeup and split finger). This group includes a slower release speed and a lower *Δ*Z value than the 4-seam. ΔX shows a positive value. These pitch classifications would be supported by our previous data that we showed the categorization for ball types among 84 skilled pitchers, based on ball speed, the direction of the spin axis, and the spin rate (34). The seven changing ball types had the characteristic that the ball was released on the outside (higher X coordinate value) to the 4-seam.

This study has some limitations. First, the data reliability, accuracy, and definition of pitch types were dependent on the PITCHf/x and TrackMan systems. These systems do not describe the definition of the origin of the 3D coordinates (X, Y, and Z axes) of the release point. Thus, we estimated the origin to be the center of the pitcher's plate and used 3D coordinates. Second, the data for release speed, spin rate, and 3D coordinates were defined at the time of the pitcher's ball release. In order to throw a fast ball, elbow flexion torque, shoulder proximal force, and elbow proximal force are important motion parameters (35). Age, height, and shoulder internal rotation strength also affect ball speed among adolescent pitchers (36). Therefore, if the kinematics data of pitching are recorded using high-speed cameras, the details of the mechanisms would be clarified. Finally, the data on 4-seam in this study was calculated by averaging over 50 balls. This average value should be changed under some conditions. For example, fatigue with increased pitch innings caused a decrease in ball speed (37–39), and ball count is also related to the pitching performance (40). Our big data included these factors.

## Conclusion

Our main findings are as follows: (1) The release points in the X, Y, and Z axes differed significantly between seven different ball types (sinker, slider, changeup, cutter, curve, split finger, and knuckle curve) and 4-seam. (2) Each of the seven changing ball types had a unique release speed, spin rate, and amount of change. (3) Eight pitch types were divided into three groups (4-seam, curve, and off-speed pitch types) depending on the characteristics of release speed, spin rate, X, Y, Z, ΔX and ΔZ values, and correlation coefficients.

## Equations

Formula 1. $\sqrt{{\left(x_{1}-x_{2}\right)}^{2}\;+\;{\left(y_{1}-y_{2}\right)}^{2}}$.

Formula 2. $\sqrt{{\left(x_{1}-x_{2}\right)}^{2}\;+\;\;{\left(y_{1}-y_{2}\right)}^{2}+{\left(z_{1}-z_{2}\right)}^{2}}$.

Formula 3.

$$\begin{array}{rl} \mathrm{Corrected}\;\mathrm{value}\;= & \;\mathrm{Average}\;\mathrm{data}\;\mathrm{of}\;\mathrm{the}\;\mathrm{corresponding}\;\mathrm{ball}\;\mathrm{type}\;\\ & -\;\mathrm{The}\;\mathrm{average}\;\mathrm{value}\;\mathrm{of}\;\mathrm{the}\;\mathrm{pitche}r^{\prime }s\;4\\ & -\mathrm{seam}\;+\;4-\mathrm{seam}\;(\mathrm{mean}).\; \end{array}$$

## Data Availability

The original contributions presented in the study are included in the article/supplementary materials, further inquiries can be directed to YH, yasu88_@hotmail.co.jp.
